# Linc00511 acts as a competing endogenous RNA to regulate VEGFA expression through sponging hsa‐miR‐29b‐3p in pancreatic ductal adenocarcinoma

**DOI:** 10.1111/jcmm.13351

**Published:** 2017-10-05

**Authors:** Xiaohui Zhao, Yimin Liu, Zhihua Li, Shangyou Zheng, Zairui Wang, Wenzhu Li, Zhuofei Bi, Liting Li, Yanhui Jiang, Yuming Luo, Qing Lin, Zhiqiang Fu, Chen Rufu

**Affiliations:** ^1^ Department of Radiotherapy Sun Yat‐sen Memorial Hospital Sun Yat‐sen University Guangzhou China; ^2^ Key Laboratory of Malignant Tumor Gene Regulation and Target Therapy of Guangdong higher Education Institutes Sun Yat‐Sen Memorial Hospital Sun Yat‐sen University Guangzhou China; ^3^ Department of Medical Oncology Sun Yat‐sen Memorial Hospital Sun Yat‐sen University Guangzhou China; ^4^ Department of Hepatobiliary Surgery Sun Yat‐sen Memorial Hospital Guangzhou China; ^5^ Department of Nephrology Armed Police Corps Hospital of Guangdong Province Guangdong China

**Keywords:** Pancreatic ductal adenocarcinoma, Competing endogenous RNA, linc00511, hsa‐miR‐29b‐3p, VEGFA

## Abstract

Pancreatic ductal adenocarcinoma (PDAC) is a lethal malignancy. Long non‐coding RNAs (lncRNAs) are important regulators in pathological processes, yet their potential roles in PDAC are poorly understood. Here, we identify a fundamental role for a novel lincRNA, linc00511, in the progression of PDAC. Linc00511 levels in PDAC tissue specimens and cell lines were examined by quantitative real‐time PCR. Corresponding adjacent non‐neoplastic tissues were used as controls. The function of linc00511 in PDAC cell lines was determined by RNA interference approach *in vitro* and *in vivo*. Fluorescence *in situ* hybridization (FISH) was used to characterize linc00511 expression in PDAC cells. Insights of the mechanism of competitive endogenous RNAs (ceRNAs) were obtained from bioinformatic analysis, luciferase assays and RIP assays. The association between the linc00511/hsa‐miR29b‐3p axis and VEGFA was verified by Western blotting assay. Immunohistochemistry was performed to evaluate the expression of VEGFA in PDAC samples. The aberrant up‐regulation of linc00511 was detected in PDAC cell lines and patient specimens compared with controls. An increase in linc00511 expression indicates the adverse clinical pathological characteristics and poor prognosis. Functionally, linc00511 depletion in PDAC cells decreased proliferation, migration, invasion and endothelial tube formation. Mechanistically, linc00511 could up‐regulate VEGFA *via* its competing endogenous RNA (ceRNA) activity on hsa‐miR‐29b‐3p. In summary, our results define an important axis controlling proliferation, invasion and tumour angiogenesis in PDAC. Linc00511 is a novel lncRNA that plays a significant regulatory role in the pathogenesis and progression of PDAC. Thus, Linc00511 represents a new prognostic biomarker to predict clinical outcome of PDAC patients after surgery and may serve as a potential therapeutic target for PDAC treatment.

## Introduction

PDAC is a highly malignant neoplasm and the fourth leading cause of death in cancer patients 1, 2. PDAC is characterized by a highly malignant phenotype that is associated with early metastasis and resistance to chemotherapy and radiation therapy 3, 4. Thus, in‐depth research of the genetic alterations and underlying molecular mechanism of PDAC is still an urgent issue.

Recently, accumulating data demonstrated that non‐coding RNAs (ncRNAs) are key regulators in both normal physiological and pathological conditions 5, 6, 7. LncRNAs which are defined as those longer than ~200 nucleotides, have been extensively characterized and it is evident that they are taking crucial roles in pathologies, including cancers 8, 9, 10, 11, 12, 13. In our previous study (GEO, http://www.ncbi.nlm.nih.gov/geo/,ID:GSE61166), microarrays were used to examine the expression profiles of mRNAs and lncRNAs in PDAC. We identified many lncRNAs aberrantly expressed between PDAC and non‐neoplastic tissues, among which a novel lncRNA linc00511 was significantly up‐regulated in PDAC samples. Linc00511, a 2.265 kb lncRNA, that maps to chromosome 17q24.3, was reported to be highly expressed in the aggressive basal‐like breast cancer subtype 14. Furthermore, Cheng‐Cao Sun *et al*. reported that linc00511 was associated with the development of non‐small cell lung cancer, wherein elevation of linc00511 expression induced proliferation and invasion. They also showed that the activity of linc00511 was due, in part, to the interaction of linc00511 with histone methyltransferase enhancer of zeste homologue 2 15. To date, little is known about its involvement in PDAC, and the biological roles of linc00511 in cancer cells also remain elusive.

Vascular endothelial growth factor A (VEGFA), a member of the PDGF/VEGF growth family, encodes a heparin‐binding protein, which exists as a disulfide‐linked homodimer. VEGFA is a proangiogenic cytokine that sustain tumour angiogenesis and limit antitumor immunity. Tumour angiogenesis is an important step in both tumour proliferation and metastasis formation. In fact, VEGFA acts as a key regulator of proliferation, survival, migration, permeability of blood endothelial cells in both physiological and pathological angiogenesis 16, 17, 18. VEGFA amplification has been implicated in more unfavourable prognostic features in colorectal cancer including advanced stage, vascular and lymphatic invasion and significantly poorer survival time 19. Meanwhile, VEGFA was also found to be markedly upregulated in other tumours. The overexpression of VEGFA is a powerful predictor of overall survival and progression for some cancers, including breast cancer, prostate cancer, lung cancer and glioma 20, 21, 22, 23, 24. Additionally, VEGFA was also demonstrated to increase the motility of PDAC cells 25. However, the expression levels and underlying mechanism of VEGFA in PDAC remain to be elucidated.

The focus of this research was to identify the roles that linc00511 plays in PDAC, and to uncover the potential mechanisms by which linc00511 exerts its oncogenic activity. We first evaluated the expression of linc00511 in PDAC and explored the clinical significance of linc00511 expression. Subsequently, both *in vitro* and *in vivo* models were utilized to demonstrate the biological roles of linc00511 on the phenotypes of pancreatic cancer cells. In particular, our results revealed that linc00511 may function as a ceRNA to mediate the expression of VEGFA through competition for hsa‐miR‐29b‐3p, hence serving as a tumour promoter in PDAC pathogenesis. Thus, linc00511 could be a promising molecular biomarker and therapeutic target for PDAC.

## Materials and methods

### Tissue collection

A total of 140 cases of fresh‐frozen pancreatic cancer tissues and corresponding adjacent non‐neoplastic pancreatic tissues were obtained from patients undergoing pancreas resection in Sun Yat‐Sen Memorial Hospital of Sun Yat‐Sen University between 2009 and 2015. Informed consent was obtained from all patients before sample collection. All specimens were immediately frozen in liquid nitrogen after removal. All patients selected met the following criteria: the histopathological diagnoses of all patients were clear and definite. All patients had received no local or systemic therapy before surgery, and exhaustive clinical‐pathologic and follow‐up data were available.

### Cell culture

PANC‐1, MIA PaCa‐2, Capan‐2, SW1990, ASPC‐1, BxPC‐3 cells were purchased from ATCC. An immortalized human pancreatic ductal epithelial cell line (HPDE6) was kindly provided by Dr. SN Zhang (Sun Yat‐Sen University, Guangdong, China). The procedures are briefly described in supplementary methods Data S1.

### RNA isolation and quantitative real‐time PCR

Total RNA was isolated from cells and tissues using TRIzol reagent (Invitrogen, San Diego, CA, USA). Quantitative real‐time PCR was used to assess linc00511, has‐miR‐29b‐3p and VEGFA expression levels. Additional details were provided in Supplementary Methods Data S1. The qRT‐PCR data were normalized using the endogenous GAPDH and U1 for linc00511. β‐actin or U6 snRNA was used as internal controls for mRNA or miRNA, respectively. The relative gene expression in cells was determined using the comparative delta‐delta CT method (2‐∆∆Ct), and the fold change in gene expression of tissues was calculated using the standard ∆∆Ct method. All the primer sequences are provided in Table S1.

### Cell transfection and virus infection

SW1990 cells and ASPC‐1 cells were transfected with 100 nM of scramble control siRNA or linc00511 siRNA#1 or linc00511 siRNA#2 purchased from GenePharma Co (Shanghai, China), using Lipofectamine 3000 reagent (Invitrogen). The stable silencing of linc00511 was performed by shRNA interference. The procedures are described in Supplementary Methods Data S1. All oligonucleotide sequences are provided in Table S1.

### Western blotting

Western blotting was performed as described in Supplementary Methods Data S1. Mouse anti‐human VEGFA polyclonal antibody (5 μg/ml, #ab1316; Abcam, Cambridge, MA, USA) was used for analysis. β‐actin (1:1000, #8226; Abcam) was used as a loading control.

### Proliferation, apoptosis, migration and invasion assay

Cell growth was evaluated *via* the Counting Kit‐8 kit (CCK‐8) assay. Apoptotic levels were assessed *via* Annexin V‐fluorescein isothiocyanate/propidium iodide (Annexin V‐FITC/PI) double staining assay. Migration was assayed using wound‐healing scrach assay. Invasion was determined *via* transwell assay. Additional details are given in Supplementary Methods Data S1.

### Production of conditioned media (CM) and tube formation assay

Transfected ASPC‐1 and SW1990 cells were cultured in DMEM containing 0.5% FBS for 48 hrs and conditioned media (CM) were collected. HUVECs were incubated in CM for 24 hrs and resuspended in respective CM. One hundred microlitres of the cell suspension (2 × 10^4^ cells) was loaded onto the surface of the polymerized Matrigel and incubated at 37°C for 16 hrs. Five randomly chosen fields were calculated and photographed (Olympus, Tokyo, Japan). Additional details are given in Supplementary Methods Data S1.

### FISH

A fragment of linc00511 designed as its probe was amplified by the upstream primer and downstream primer and then cloned into the pMD‐18T vector. The fragment was utilized and labelled with (FITC)‐UTP (Roche, Basel, Switzerland) using a mMESSAGE T7 Ultra *In Vitro* Transscription kit (Life Technologies, Grand Island, NY, USA). Slides were hybridized with probes overnight, washed thrice with 50% formamide/2 × saline‐sodium citrate (SSC) and thrice with 2 × SSC at 45°C for 5 min. Glass slides were mounted on slides using a fluorescence mounting medium from China Biyun (Beijing, China). Cells were observed and photomicrographed using a fluorescence microscope (Olympus).

### Vector preparation

Linc00511 cDNA was cloned into the vector pcDNA3.1. Human microRNA precursors with about 80 bp of flanking sequences in both sides were amplified and cloned into the modified Pll3.7 vector (Invitrogen) in order to express miRNAs. The full length of linc00511 and the 3′UTR of VEGFA were amplified and inserted into pMIR‐REPORT vectors. The mutant linc00511 and 3′UTR of VEGFA constructs were generated using the QuikChange site‐directed mutagenesis kit (Stratagene, La Jolla, CA, USA). Has‐miR‐29b‐3p mimic and mimic control as well as has‐miR‐29b‐3p inhibitor were synthesized by GenePharma.

### Luciferase report assay

ASPC‐1 and SW1990 cells were co‐transfected with pMIR‐linc00511, pMIR‐linc00511‐Mut, pMIR‐VEGFA‐3UTR or pMIR‐VEGFA‐Mut‐3UTR, along with the pRL‐TK control vector used as an internal control and has‐miR‐29b‐3p mimic or mimic control. The luciferase reporter assay was conducted using a double‐luciferase assay system (Promega, San Luis Obispo, CA, USA).

### RNA binding protein immunoprecipitation assay

RIP assay was conducted using the Magna RIP RNA‐Binding Protein Immunoprecipitation Kit (Millipore, Bedford, MA, USA) according to the manufacturer's protocol. Additional details are given in Supplementary Methods Data S1.

### Xenograft study

All animal studies were performed in accordance with the National Academy of Science Guide for the Care and Use of Laboratory Animals and United Kingdom Co‐ordinating Committee on Cancer Research guidelines. All experimental protocols were approved by the committee on the Ethics of Animal Experiments of the Sun Yat‐Sen University. The procedures of tumour formation were given in Supplementary Methods Data S1.

### Immunohistochemistry study

Patient samples of primary carcinomas and the xenograft tumour specimens from nude mice were stained for VEGFA or Ki67, respectively. Ki67 staining was quantified by determining the proportion of Ki67‐positive cells. For VEGFA evaluation, we adopted a scoring criterion previously reported by Ohara *et al*. 26. The procedures of immunohistochemistry staining and evaluation were given in Supplementary Methods Data S1.

### Statistical analysis

The chi‐square test for non‐parametric variables and Student's *t*‐test for parametric variables were used (two‐tailed). Differences in patient survival were assessed using the Kaplan–Meier method. A ROC curve was established to examine the diagnostic value of linc00511. The AUC was utilized to assess the predictive power. To assess the relative risk for each factor, univariate and multivariate Cox regression analysis were performed. All tests were two‐sided, and *P* < 0.05 was considered statistically significantly. Analysis was performed using SPSS software (IBM, Armonk, NY, USA).

## Results

### Linc00511 is up‐regulated in PDAC and is associated with cancer progression and poor prognosis

**Figure 1:**
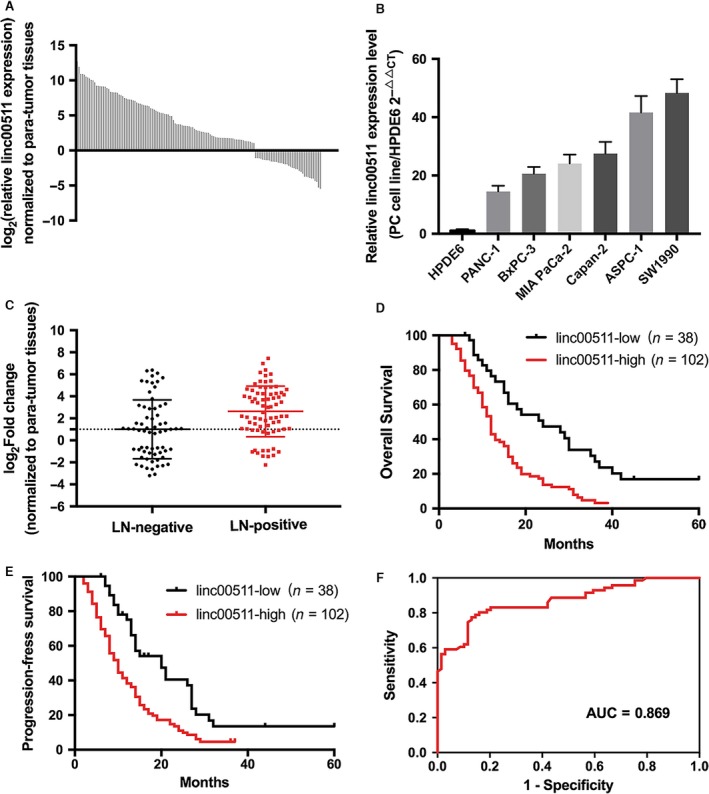
High Linc00511 expression in PDAC correlates with poor patient prognosis. (**A**) Linc00511 expression level in 140 paired PDAC tissues and corresponding adjacent non‐neoplastic tissues was examined *via* quantitative reverse transcription‐polymerase chain reaction (qRT‐PCR). GAPDH and U1 were used as internal controls. Data are represented as log_2_ fold changes (cancer/normal), and defined as ‘<‐1’ for underexpression and ‘>1’ for overexpression. The patients were divided into a low‐linc00511 expression group (*n* = 38) and a high linc00511 expression group (*n* = 102) according to whether linc00511 was up‐ or down‐regulated in their tumour tissue samples compared with the corresponding adjacent noncancerous tissue samples. (**B**) Linc000511 expression was evaluated in six pancreatic cancer cell lines compared with immortalized human ductal epithelial cells by qRT‐PCR. Linc00511 mRNA levels were normalized to GAPDH and U1. Data represent the mean ± S.D. From three independent experiments. (**C**) Linc00511 expression in the lymph node metastasis‐negative group and metastasis‐positive group. (**D**,** E**) Kaplan–Meier survival curves showing different overall survival and progression‐free survival in groups of PDAC patients with low and high linc00511 expression in tumours. (**F**) ROC curve analysis showing the performance of linc00511 in predicting tumour progression within 1 year after surgical resection.

To investigate the oncogenic role of linc00511 in PDAC progression, we examined the expression of linc00511 in human PDAC tissues and PDAC cell lines by quantitative real‐time PCR. As shown in Figure 1A and B, linc00511 was up‐regulated in most PDAC tissues compared with the adjacent non‐tumour tissues. Linc00511 was overexpressed in all of the five pancreatic cancer‐derived cell lines (PANC‐1, Capan‐2, MIA PaCa‐2, BxPC‐3 and SW1990) compared with the non‐tumoral pancreatic cell line, HPDE6. Among the six cell lines, linc00511 are relative higher expressed in ASPC‐1 and SW1990 cells; therefore, we chose ASPC‐1 and SW1990 cells to perform the following experiments. These data suggest that linc00511 is overexpressed in PDAC.

Linc00511 expression levels in tumour tissues were categorized as low or high depending on whether linc00511 expression was up‐ or down‐regulated compared with the corresponding adjacent non‐tumoral tissue samples. Table 1 shows that linc00511 overexpression was associated with lymphatic metastasis (Fig. 1C) and early recurrence. The other clinical characteristics showed no statistical relationship with linc00511 expression. Univariate analysis of overall survival revealed that lymph node metastasis (*P* < 0.001), early recurrence (*P* < 0.001) and linc00511 expression (*P* < 0.001) were prognostic indicators (Table 2). Multivariate analysis indicated that linc00511 expression and early recurrence (*P* < 0.001)were independent prognostic indicators for overall survival of patients with PDAC. Kaplan–Meier survival analysis showed that patients with low linc00511 expression had significantly increased overall survival and progression‐free survival as compared to patients with high expression (Fig. 1D and E). We also constructed a ROC (receiver operating characteristic) curve analysis (Fig. 1F). For predicting progression within 1 year, the area under the ROC curve was 0.869 (*P* < 0.0001) with an optimal cut‐off point of 5.94 (sensitivity =77.47%, specificity = 86.96%).

**Table 1:** Correlation of linc00511 expression and clinic‐pathological factors of patients with PC

Characteristics	No. of patients	Linc00511		
		Low	High	*P* value
Total	140	38	102	
Age (years)				
<60	71	17	54	0.388
≥60	69	21	48	
Gender				
Male	78	20	58	0.654
Female	62	18	44	
Differentiation				
Well	50	16	34	0.359
Moderate	40	12	28	
Poor	50	10	40	
T stage				
T1	50	17	33	0.267
T2	35	10	25	
T3	55	11	44	
N stage				
N0	66	27	39	**0.001**
N1	74	11	63	
Neural invasion				
Negative	68	15	53	0.189
Positive	72	23	49	
Early recurrence				
No	69	29	40	<**0.001**
Yes	71	9	62	

Bold values are statistically significant (*P *<* *0.05).

**Table 2:** Univariate and multivariate Cox regression of prognostic factors for overall survival in pancreatic cancer

Parameter	Univariate analysis			Multivariate analysis		
	HR	95% CI	*P*	HR	95%	*P*
Age(<60 *versus* ≥60)	1.042	0.946–1.148	0.404			
Gender(Female *versus* Male)	0.946	0.858–1.042	0.259			
Differentiation(well *versus* moderate *versus* poor)	1.053	0.995–1.114	0.073			
T stage(T1 *versus* T2 *versus* T3)	1.045	0.988–1.105	0.127			
N stage(N0 *versus* N1)	1.407	1.276–1.552	**<0.001**	1.076	0.971–1.193	0.162
Neural invasion(negative *versus* positive)	1.089	0.988–1.200	0.085			
Early recurrence	3.782	3.392–4.216	**<0.001**	2.616	2.330–2.936	**<0.001**
Linc00511 expression(low *versus* high)	3.097	2.768–3.446	**<0.001**	2.258	1.989–2.563	**<0.001**

Bold values are statistically significant (*P* < 0.05).

### Linc00511 promotes the proliferation of PDAC cells *in vitro* and *in vivo*

**Figure 2:**
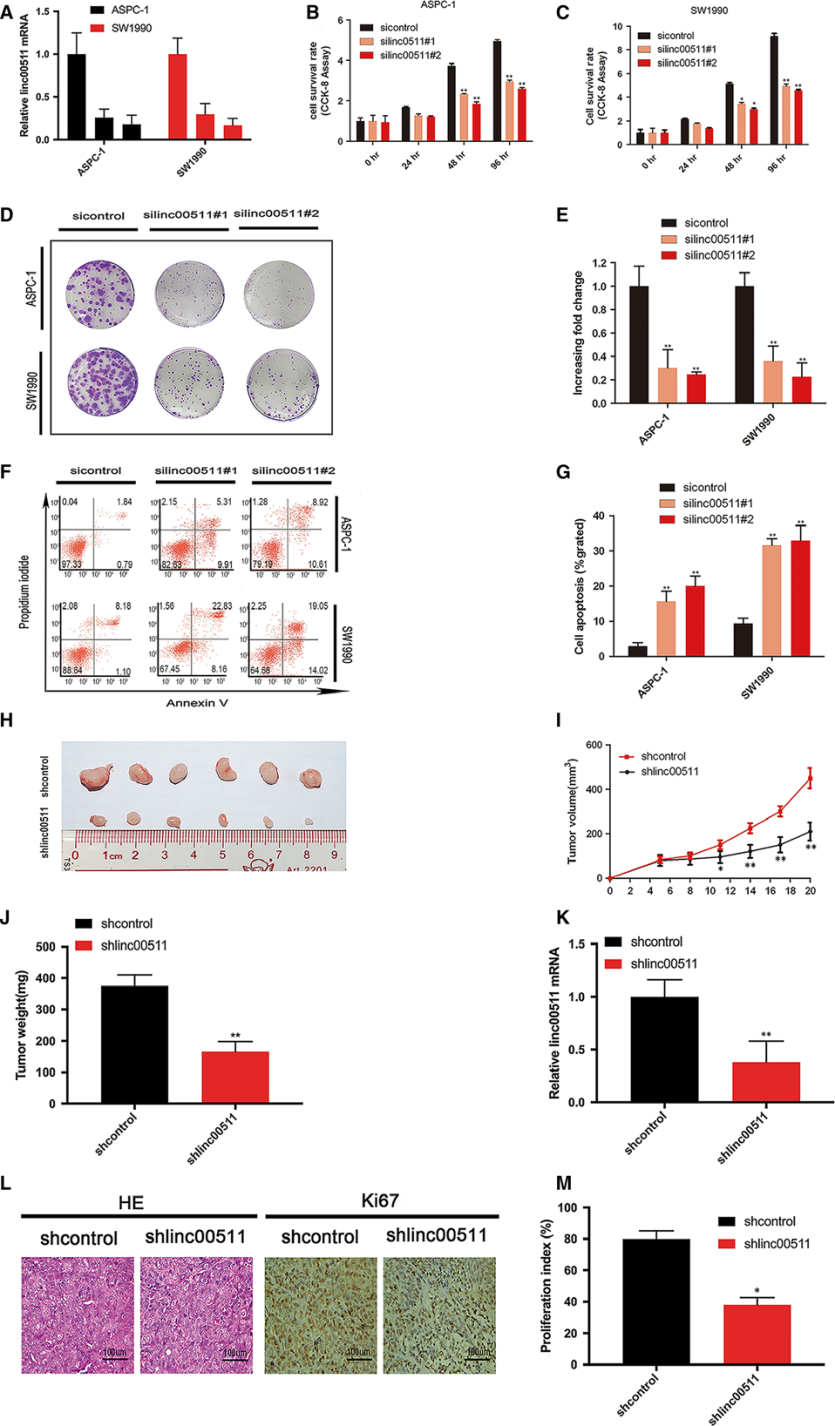
Effect of linc00511 on the PDAC cell growth *in vitro* and *in vivo*. (**A**) ASPC‐1 and SW1990 cells were transfected with control siRNA or siRNAs against linc00511, and linc00511 expression was subsequently determined by qRT‐PCR. (**B**,** C**) The influence of linc00511 knockdown on cancer cell lines growth was evaluated CCK‐8 assays. (**D**,** E**) The influence of linc00511 knockdown on cancer cell lines growth was by colony formation assays. (**F**,** G**) The influence of linc00511 knockdown on the cell apoptosis was evaluated by flow cytometry. (**H**) Images of tumours removed from all mice after 20 days after injection of SW1990 cells infected with shRNA targeting linc00511 or control shRNA. (**I**) Tumour growth curve. Point indicate mean (*n* = 6) and bars indicate S.D. (**J**) Tumour weights were evaluated and shown as the mean weights ± S.D. when the tumours were harvested. (**K**) Linc00511 levels in tumour tissues from shlinc00511 SW1990 group and shcontrol group were determined by qRT‐PCR. (**L**,** M**) Representative images (×200) of H&E and IHC staining of the tumour. The IHC staining showed that inhibition of linc00511 impaired the proliferation of PDAC *in vivo*, as indicated by the expression of Ki67. The proliferation index was quantified by determining the proportion of Ki67‐positive cells. The results are presented as mean ± S.D. of values obtained in at least three independent experiments. *P* < 0.01, Student's *t*‐test.

To investigate whether linc00511 has a role in the pathogenesis of PDAC, we evaluated the consequences of linc00511 knockdown on tumour cell physiology by evaluating cell proliferation, apoptosis and invasive behaviour. We employed siRNA to specifically silence the expression of linc00511 in ASPC‐1 and SW1990 cells (Fig. 2A). The proliferation index measurement at 48 hrs after siRNA delivery in ASPC‐1 and SW1990 cells showed a significant reduction in both cell lines as compared to controls (Fig. 2B and C). The colony formation assay also revealed that linc00511 downregulation greatly decreased the colony numbers of PDACs (Fig. 2D and E). To determine whether PDAC cell proliferation was influenced by apoptosis, we performed flow cytometric analysis. The results revealed that knockdown of linc00511 could obviously induce cell apoptosis (Fig. 2F and G). SW1990 cells stably transfected with shRNA targeting linc00511 or empty vector was subcutaneously injected into nude mice. Consistent with the *in vitro* results, tumour growth in the shlinc00511 group was significantly diminished relative to the shcontrol group (Fig. 2 H, I and J). According to the results of qPT‐PCR analysis, we verified the depletion of linc00511 in the xenotransplanted tumours from the shlinc00511 group (Fig. 2K). Moreover, immunostaining indicated that tumours from the shlinc00511 group showed a reduced positive rate of Ki67 than the shcontrol group (Fig. 2L and M).

### Linc00511 promotes tumour migration, invasion and tumour angiogenesis in PDAC

**Figure 3:**
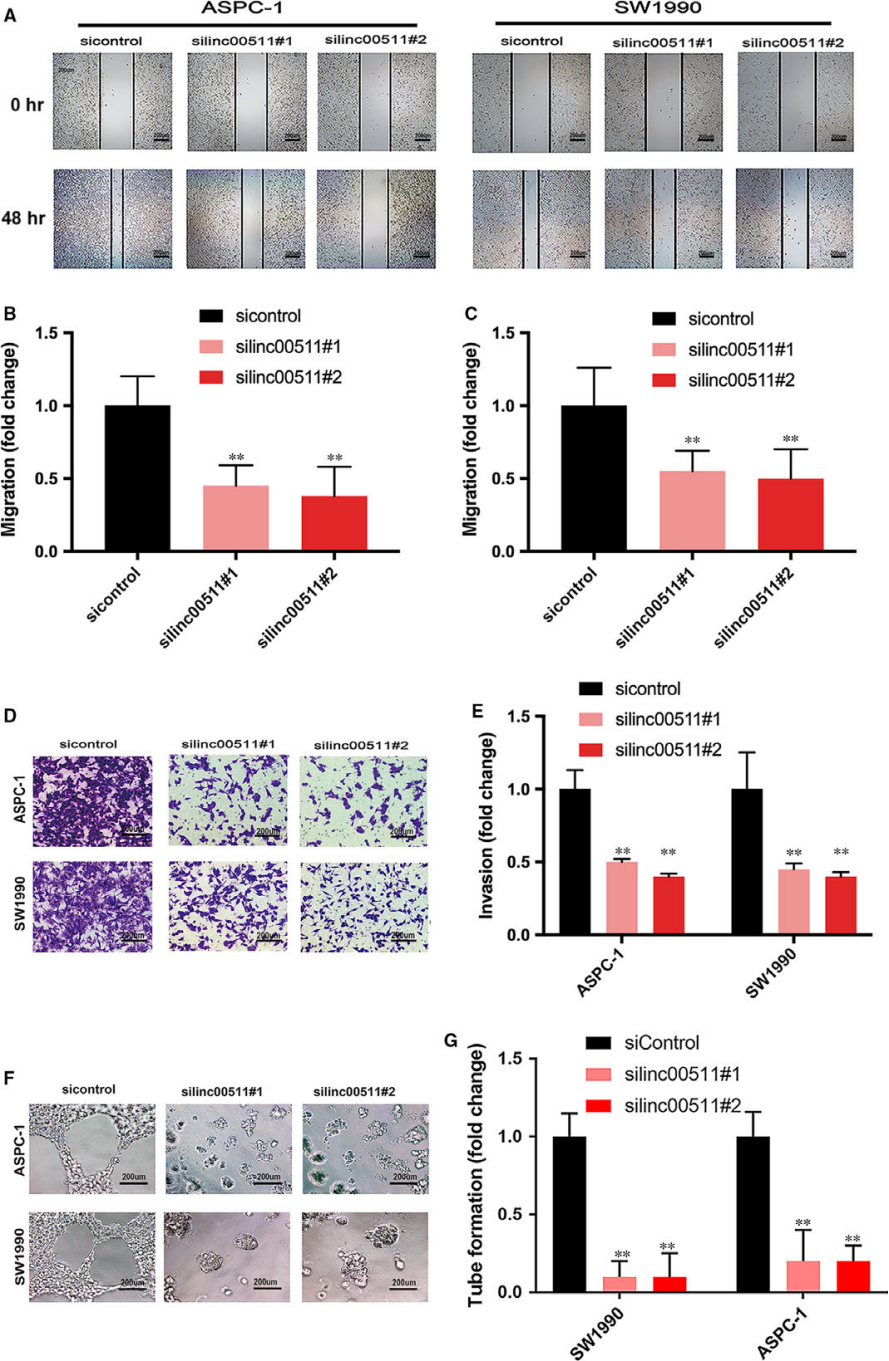
Effect of linc00511 on the PDAC cell migration, invasion and tumour angiogenesis. (**A**,** B**,** C**) Cell motility was detected after transfection with siRNA targeting linc00511 or control siRNA by wound healing assays. (**D**,** E**) Cell invasion was determined after transfection with siRNA targeting linc00511 or control siRNA using transwell assays. (**F**,** G**) Tube formation of HUVECs was measured after 16 hrs treatment with conditioned medium from linc00511‐downregulated ASPC‐1 and SW1990 cells and their respective controls.

We evaluated whether the depletion of linc00511 affected PDAC cell migration and invasion. Wound healing assay indicated that knockdown of linc00511 in ASPC‐1 and SW1990 cells dramatically decreased the ability of the cells to migrate (Fig. 3A, B and C). In addition, transwell assay demonstrated that the inhibition of linc00511 markedly attenuated the invasion of ASPC‐1 and SW1990 cells (Fig. 3D and E).

We evaluated the ability of tube formation. The result showed that relative tubule length and number of branches were decreased in silinc00511 group compared with that in sicontrol group (Fig. 3F and G).

### Linc00511 acts as a sponge for hsa‐miR‐29b‐3p in PDAC cells

**Figure 4:**
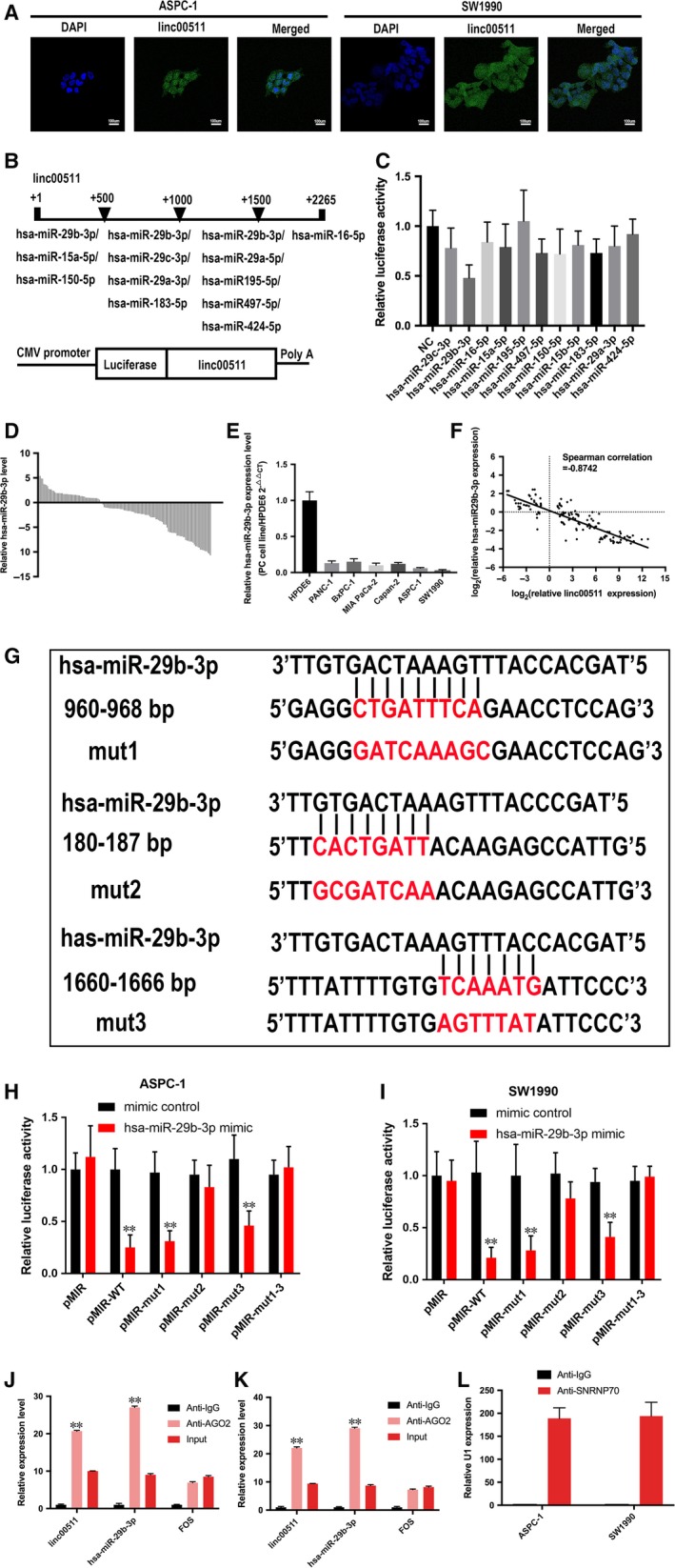
The interaction of linc00511 with hsa‐miR‐29b‐3p. (**A**) The *in situ* expression of linc00511 in PDAC cells. The green fluorescent signals are from the FITC‐linc00511 probe. The blue fluorescent signal is from nuclear DNA counterstained with DAPI. (**B**) The 11 putative miRNA binding sites in the linc00511 sequence. The linc00511 cDNA containing the putative miRNAs was cloned downstream of the luciferase gene and named Rluc‐linc00511. (**C**) RLuc‐linc00511 was co‐transfected into ASPC‐1 cells with the 11 various miRNA‐coding plasmids. (**D**) Has‐miR‐29b‐3p expression levels in 140 paired PDAC tissues and corresponding adjacent non‐neoplastic tissues were examined *via* quantitative reverse transcription‐polymerase chain reaction (qRT‐PCR). U6 were used as internal controls. The data shown are fold changes of has‐miR29b‐3p expression in PDAC cases normalized to paired normal adjacent tissues. (**E**) Linc000511 expression was evaluated in six pancreatic cancer cell lines compared with immortalized human ductal epithelial cells by qRT‐PCR. U6 were used as internal controls. (**F**) Correlation scatterplot of relative linc00511 and has‐miR‐29b‐3p levels. Data are represented as log_2_ fold changes in PDAC tissues relative to paired normal adjacent tissues. (**G**) The presentation of hsa‐miR‐29b‐3p's binding sites on linc00511. (**H**,** I**) Luciferase activity in ASPC‐1 and SW1990 cells co‐transfected with hsa‐miR‐29b‐3p mimic or mimic control and luciferase reporters containing nothing (pMIR), wild‐type linc00511 (pMIR‐WT) or diverse mutant linc00511 as indicated. (**J**,** K**,** L**) RIP assay was performed utilizing input from cell lysate, normal mouse IgG or anti‐Ago2. (**J**,** K**) levels of linc00511, has‐miR‐29b‐3p, and FOS RNA were presented as fold enrichment in Ago2 relative to IgG immunoprecipitates. (**L**) relative RNA levels of U1 snRNA in SNRNP70 relative to IgG immunoprecipitates. The results are presented as mean ± S.D. of values obtained in at least three independent experiments. *P* < 0.01, Student's *t*‐test.

We performed FISH to identify the distribution of linc00511 in PDAC cells. The results showed that linc00511 was abundant in both cytoplasm and nucleus (Fig. 4A). Inspired by the ‘ceRNAs’ regulatory network and emerging evidence suggests that lncRNAs may participate in this regulatory circuitry, we explored the function and mechanism of linc00511 of the cytoplasmic form. We utilized the webserver StarBase v2.0(http://starbase.sysu.edu.cn) to predict potential lncRNA‐miRNA interactions. A cohort of 11 potential miRNAs that could interact with linc00511 was predicted. To further search for specific target miRNA of linc00511 in PDAC cells, the linc00511 cDNA was cloned downstream of the luciferase gene and named RLuc‐linc00511 (Fig. 4B), then transfected together with various miRNA‐coding plasmids. rno‐miR‐344 acts as a negative control. The results demonstrated that luciferase activity was reduced by 52% compared with the empty vector control when has‐miR‐29b‐3p were expressed (Fig. 4C). Then, we evaluated the expression of has‐miR‐29b‐3p in 140 paired resected samples and the presented panel of cell lines by qRT‐PCR. Has‐miR‐29b‐3p was downregulated in PDAC tissues (Fig. 4D) and cell lines (Fig. 4E). Spearman's analyses performed using qRT‐PCR expression data indicate that linc00511 and has‐miR‐29b‐3p have a high correlation score in PDAC tissues (Fig. 4F). Thus, we chose has‐miR‐29b‐3p as a model miRNA for further studies. To determine whether hsa‐miR‐29b‐3p was directly bound to linc00511, we constructed a series of luciferase reporters containing the wild‐type linc00511 (pMIR‐WT), or a series of mutant linc00511 with mutations of a single (pMIR‐mut1, 2, 3) or all 3 predicted hsa‐miR‐29b‐3p binding sites (pMIR‐ 1‐3) (Fig. 4G). We found that transfection of hsa‐miR‐29b‐3p mimic into ASPC‐1 and SW1990 cells reduced the luciferase activity of the wild‐type linc00511 reporter (pMIR‐WT) but not empty vector or all hsa‐miR‐29b‐3p site mutant reporter vector, suggesting the binding of hsa‐miR‐29b‐3p to these sites. Among them, the second hsa‐miR‐29b‐3p binding site seemed to be the strongest interaction site, because the mutation of this sequence almost abolished the effects of hsa‐miR‐29b‐3p on luciferase activity (Fig. 4H and I). As RISC is indispensable for siRNA and miRNA‐mediated gene silencing, we hypothesized that linc00511 and has‐miR‐29b‐3p might be in the same RISC complex. We performed an RNA binding protein immunoprecipitation assay using antibody against Ago2, a key component of RISC. The precipitated RNA levels were analysed by real‐time PCR and results showed that both linc00511 and hsa‐miR‐29b‐3p were significantly enriched in Ago2‐containing miRNPs relative to control immunoglobulin G (IgG) immunoprecipitates. Successful immunoprecipitation of Ago2‐associated RNA was verified by qRT‐PCR, utilizing RIP primers against human FOS included in the RIPAb+ Ago2 kit (Fig. 4J and K). Furthermore, anti‐SNRNP70 was used as a positive control for the RIP procedure, and U1 snRNA was also detected at a greater level than that of anti‐IgG (Fig. 4L). Altogether, these results demonstrate that linc00511 serves as a natural sponge for hsa‐miR‐29b‐3p.

### Linc00511 induces the expression of an endogenous hsa‐miR‐29b‐3p target, VEGFA

**Figure 5:**
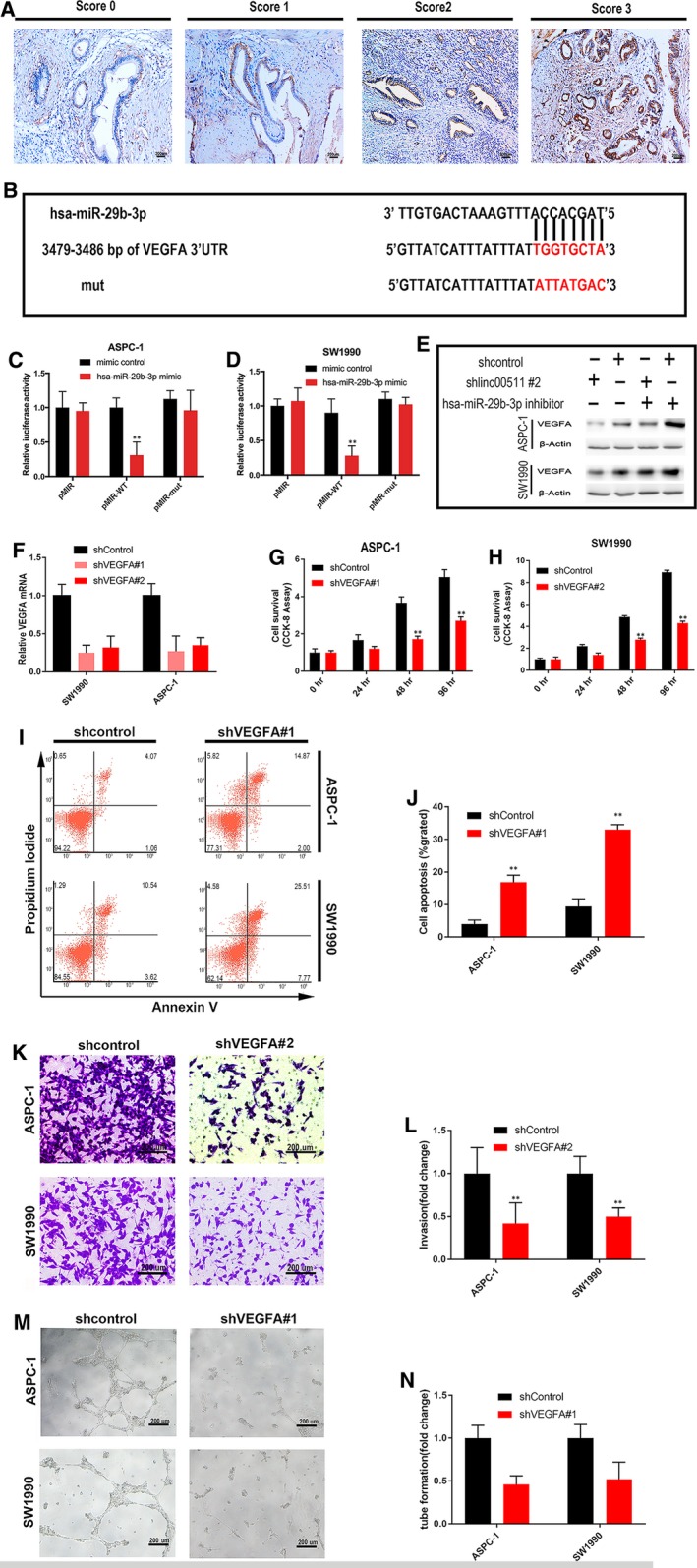
Linc00511 regulates expression of endogenous hsa‐miR‐29b‐3p targeting VEGFA. (**A**) Representative images of VEGFA staining in PDAC tissues (score 0–score 3). (**B**) Alignment of potential hsa‐miR‐29b‐3p‐binding site in the 3′‐UTR of the VEGFA mRNA. (**C**,** D**) Luciferase activity in ASPC‐1 and SW1990 cells co‐transfected with hsa‐miR‐29b‐3p mimic or mimic control and luciferase reporters containing nothing (pMIR), wild‐type VEGF‐A (pMIR‐WT) or mutant VEGF‐A as indicated. (**E**) The effect of linc00511 and hsa‐miR‐29b‐3p on VEGFA was determined using Western blot analysis. (**F**) ASPC‐1 and SW1990 cells were infected with control shRNA or shRNAs against VEGFA, and VEGFA expression was subsequently determined by qRT‐PCR. (**G**,** H**) The influence of VEGFA knockdown on cancer cell lines growth was evaluated CCK‐8 assays. (**I**,** J**) The influence of VEGFA knockdown on the cell apoptosis was evaluated by flow cytometry. (**K**,** L**) Cell invasion was determined after infection with shRNA targeting VEGFA or control shRNA using transwell assays. (**M**,** N**) Tube formation of HUVECs was measured after 16 hrs treatment with conditioned medium from VEGFA‐downregulated ASPC‐1 and SW1990 cells and their respective controls.

PDAC overexpress VEGFs which play multi‐faceted roles in stimulating neo‐angiogenesis and tumour growth, invasion, metastasis and decreased survival on the vasculature and on cancer cells 27, 28. Our research showed that linc00511 promotes tumour angiogenesis in PDAC. Thus, among the numerous targets of hsa‐miR‐29b‐3p, we focused on VEGFA that is a vital member of the VEGF family. Immunostaining was applied to examine the expression of VEGFA in 140 PDAC samples. As described in Methods, the expression of VEGFA was evaluated in terms of intensity and percentage, and finally expressed as a score of 0, 1, 2, or 3. Representative photographs for immunostaining are shown in Figure 5A. In total, 68.6% (96/140) patients had high VEGFA expression (IHC score ≥2+). Fluorescent reporter assay showed that VEGFA was also negatively mediated by hsa‐miR‐29b‐3p in PDAC cells (Fig. 5B, C and D). Western blot showed that the silencing of linc00511 significantly decreased the protein expression of VEGF‐A and hsa‐miR‐29b‐3p knockdown markedly rescued the down‐regulation of VEGF‐A induced by linc00511 knockdown (Fig. 5E). Altogether, these results demonstrate that there is competition for hsa‐miR‐29b‐3p between linc00511 and VEGF‐A.

To further determine whether linc00511 promotes PDAC progression by regulating VEGFA, we used shRNAs to silence the expression of VEGFA in ASPC‐1 and SW1990 cells (Fig. 5F). VEGFA knockdown also decreased PDAC cell proliferation (Fig. 5G and H), invasion (Fig. 5K and L) and angiogenesis (Fig. 5M and N) and increased the sensitivity of the cells to apoptosis (Fig. 5I and J). Our results demonstrate that the regulatory function of linc00511 in PDAC biology acts, at least in part, by regulating VEGFA.

## Discussion

The discovery of various lncRNAs in humans has dramatically changed our comprehension of the mechanism of cancer. Our data demonstrated that linc00511 was up‐regulated in PDAC samples compared with adjacent non‐tumoral samples and significantly associated with lymph node metastasis, early recurrence and poor patient survival. In the light of our *in vitro* data that knockdown of linc00511 impaired tumour proliferation, concomitant with induction of cell apoptosis. Furthermore, the growth promoting effect was also confirmed *in vivo*. In addition to proliferation and apoptosis, we next evaluated the influence of linc00511 on tumour cell metastasis. Knockdown of linc00511 blocked migration, invasion and angiogenesis of PDAC cells *in vitro*. These results suggested that linc00511 might serve as a potential diagnostic biomarker or therapeutic target in PDAC.

ceRNA are RNA transcripts which can communicate with each other by reducing targeting concentration of micro‐RNA (miRNA) with the derepression of other messenger RNAs (mRNAs) having the common miRNA response elements (MREs) 29, 30, 31. The finding of ceRNA markedly enhances our comprehension of cancer molecular mechanism. Emerging evidence demonstrates that endogenous lncRNA may participate in post‐transcriptional regulation by interfering with the miRNA pathways, by functioning as ceRNAs 29, 30, 32, 33, 34. For example, HULC lncRNA serves as a ceRNA of the protein coding gene PRKACB by modulating miR‐372 availability 35. LncRNA‐ATB blocks miR‐200 family and then induced ZEB1 and ZEB2 expression in hepatocellular carcinoma 36. Additionally, lincROR works as a ceRNA, effectively becoming a sink for miR‐145, thus activating the derepression of core transcription factors Nanog, Oct4 and SOX2 in human embryonic stem cell self‐renewal 37. Information on subcellular localization of lncRNAs can provide critical information regarding a possible function as nuclear‐restricted epigenetic regulator or cytoplasmatic ceRNA. Linc00511 has been reported mainly locating in nucleus, and function as an oncogene in non‐small cell lung cancer *via* binding to EZH2 15. In our present study, we found that linc00511 was abundant both in cytoplasm and nucleus in PDAC cells. HOTAIR, a well‐known molecule in the field of tumour biology, is also located in both the nucleus and cytoplasm 38. It is evident that nuclear HOTAIR can target polycomb repressive complex 2, altering H3K27 methylation and gene expression patterns across the genome 39. Recent work reported that cytoplasm HOTAIR can function as a ceRNA to mediate the expression of human epithelial growth factor receptor 2(HER2) through competition for miR‐331‐3p 40. Herein, we explored whether linc00511 in the cytoplasm acts as a miRNA sponge using the bioinformatics analysis to investigate the potential interactions between them and choose has‐miR‐29b‐3p as a model miRNA to further studies, with a particular focus on the target gene VEGFA. Our research verified that has‐miR‐29b‐3p can bind to linc00511 directly by the putative miRNA response element in PDAC cells. Moreover, the oncogenic role of linc00511 was reinforced by inducing the expression of VEGFA. These results highlight the molecular axis comprising linc00511, hsa‐miR‐29b‐3p and VEGFA as a key player in PDAC progression.

microRNAs (miRNAs) defined as non‐coding RNAs 18‐200 nucleotides in length, modulate biological progresses by targeting mRNAs resulting in their inactivation by transcriptional control or degradation 41, 42. As a special one among them, miR‐29b has become a hot topic. miR‐29b includes miR‐29b‐1 from chromosome region 7q32 and miR‐29b‐2 from 1q32. Mature miR‐29b is therefore encoded by two different precursor stem sequences. Although the sequences of the two precursor stem sequences are distinct, the mature miR‐29bs resulting from these two stem structures are identical 43, 44. miR‐29b is gaining prominence because of its emerging roles in the regulation of multiple physiological and pathological progresses, including cell cycle and growth, apoptosis, senescence, differentiation, metastasis, immune regulation, DNA methylation and regulation of extracellular matrix 45, 46, 47, 48, 49, 50, 51. Moreover, its involvement in a variety of tumour types has also been confirmed. miR‐29b, acting as a tumour suppressor in cancer, has been correlated with prostate, gastric, lung and breast cancer 52, 53, 54, 55. VEGFA is essential for tumour proliferation, invasiveness and metastasis 56, 57. MiR‐29b regulation of VEGFA expression has been verified in various tumours. Chou *et al*. 58 suggested that the miR‐29b/VEGFA axis was significantly correlated with metastasis and angiogenesis in breast cancer. Szczyrba *et al*. 54 demonstrated that induced VEGF protein expression *via* miR‐29b inhibitors was involved in prostate carcinogenesis. Our data further disclosed that hsa‐miR‐29b‐3p was inhibited by linc00511 directly, leading to the increase of level of VEGFA in PDAC.

In summary, our results demonstrate that the linc005/hsa‐miR29b‐3p/VEGFA axis plays a critical role in the tumorigenesis and development of PDAC. A deeper characterization of the accurate role of this molecular axis in the pathogenesis of PDAC will add our comprehension of the biological basis of cancer progression and may lead to the development of a predictive diagnostic marker and therapeutic strategy for PDAC.

## Conflict of interest

There is no conflict of interest.

## Supporting information


**Table S1** Oligonucleotide sequences for this study.

**Data S1**. Supplementary methods.
