# Supplementary material for: Linc00511 acts as a competing endogenous RNA to regulate VEGFA expression through sponging hsa‐miR‐29b‐3p in pancreatic ductal adenocarcinoma
Source: J Cell Mol Med. 2017 Oct 5;22(1):655–67. doi: 10.1111/jcmm.13351 (PMC5742682; doi:10.1111/jcmm.13351)
Supplement: Supplementary file 1 — Table S1 Oligonucleotide sequences for this study. [file JCMM-22-655-s001.docx]

**Supplementary tables**

**Table S1 Oligonucleotide sequences for this study.**

| **Name** | **Forward (+),**  **Reverse(-)** | **Sequence** |
| --- | --- | --- |
| **qPCR-Primer** |  |  |
| Linc00511 | + | 5’-AGAGTTTCGCTCAGCTGCTT-3’ |
|  | - | 5’-GGTGTGAGCCAGTCCATTCA-3’ |
| hsa-miR-29b-3p | + | 5’-CTCAACTGGTGTCGTGGAGTCGGCAATTCAGTTGAGAACACTGA-3’ |
|  | - | 5’-ACACTCCAGCTGGGTAGCACCATTTGAAATC-3’ |
| VEGFA | + | 5’-CTGTCTAATGCCCTGGAGCC-3’ |
|  | - | 5’-ACGCGAGTCTGTGTTTTTGC-3’ |
| GAPDH | + | 5’-CTCTGCTCCTCCTGTTCGAC-3’ |
|  | - | 5’-ACCAAATCCGTTGACTCCGA-3’ |
| U1 | + | 5’-CAGGGCGAGGCTTTATCCA-3’ |
|  | - | 5’-GCAGGGGTCAGCACATCC-3’ |
| U6 |  | 5’-CTCGCTTCGGCAGCACA-3’ |
|  |  | 5’-AACGCTTCACGAATTTGCGT-3’ |
| **Primers for subcloning and plasmid construction** |  |  |
| pCDNA3.1-Linc00511 | + | 5’-CCCAAGCTTAGACATGGCCCAAGGAATGG -3’ |
|  | - | 5’-CCGGAATTCATGAAGCCCAGTGTAAGCCC -3’ |
| pMIR-Linc00511 | + | 5’-CCCAAGCTTAGATCCAGCCTGTTTGGACG -3’ |
|  | - | 5’- CGACGCGTGCCCCACGTTTCCACTTTTC -3’ |
| pMIR-VEGFA-3'UTR | + | 5’-CCCAAGCTTCAGCGCCATTCTACCCACTC -3’ |
|  | - | 5’-CGACGCGTCGTGCAAGTTGCTTAGGTCG -3’ |
| pLL3.7-pre-miR-29b-3p | + | 5’-CTAGCTAGCCAGTGCTGCAATGATACCGC -3’ |
|  | - | 5’-CCGGAATTCGGGAACCGGAGCTGAATGAA -3’ |
| pLL3.7-pre-miR-183-5p | + | 5’-CTAGCTAGCCAGAGTGTGACTCCTGTTCTGT-3’ |
|  | - | 5’-CCGGAATTCTCTGTTTATGGCCCTTCGGTA-3’ |
| pLL3.7-pre-miR-195-5p | + | 5’-CTAGCTAGCAGCTTCCCTGGCTCTAGCA-3’ |
|  | - | 5’-CCGGAATTCCTGGAGCAGCACAGCCAATA-3’ |
| pLL3.7-pre-miR-497-5p | + | 5’-CTAGCTAGCACACTGTGGTTTGTACGGCA-3’ |
|  | - | 5’-CCGGAATTCCTCCCCCACCCTCGCTCTAA-3’ |
| pLL3.7-pre-miR-424-5p | + | 5’-CTAGCTAGCAGGGGATACAGCAGCAATTCA-3’ |
|  | - | 5’-CCGGAATTCACCTTCTACCTTCCCCACGA-3’ |
| pLL3.7-pre-miR-16-5p | + | 5’-CTAGCTAGCGGGGTAGCAGCACGTAAAT-3’ |
|  | - | 5’-CCGGAATTCTGTCGTGGAGTCGGCAATTG-3’ |
| pLL3.7-pre-miR-29a-3p | + | 5’-CTAGCTAGCCGACCTTCTGTGACCCCTTAG-3’ |
|  | - | 5’-CCGGAATTCTCATGGTGCTCTTCCCCAT-3’ |
| pLL3.7-pre-miR-29a-5p | + | 5’-CTAGCTAGCCCCTCCGACCTTCTGTGAC-3’ |
|  | - | 5’-CCGGAATTCTGGTGCTCTTCCCCAATCAT-3’ |
| pLL3.7-pre-miR-29c-3p | + | 5’-CTAGCTAGCACACAGGCTGACCGATTTCT-3’ |
|  | - | 5’-CCGGAATTCCCCCCTACATCATAACCGATTTC-3’ |
| pLL3.7-pre-miR-150-5p | + | 5’-CTAGCTAGCCTGTCTCCCAACCCTTGTACC-3’ |
|  | - | 5’-CCGGAATTCGTCCCCAGGTCCCTGTCC-3’ |
| pLL3.7-pre-miR-15a-5p | + | 5’-CTAGCTAGCCCTTGGAGTAAAGTAGCAGCAC-3’ |
|  | - | 5’-CCGGAATTCGAGGCAGCACAATATGGCCT-3’ |
| **siRNA** |  |  |
| silinc00511 #1 | + | 5’-GACUGAAUGUGGUUCCAGAdTdT-3’ |
|  | - | 5’-UCUGGAACCACAUUCAGUCdTdT-3’ |
| silinc00511 #2 | + | 5’-GGACUGAAUGUGGUUCCAGdTdT-3’ |
|  | - | 5’-CUGGAACCACAUUCAGUCCdTdT-3’ |
| sicontrol | + | 5’-UGCAAUGAGUUAUCUCGCAdTdT-3’ |
|  | - | 5’-UGCGAGAUAACUCAUUGCAdTdT-3’ |
| **shRNA** |  |  |
| shlinc00511 | + | 5’-GATCCGGACUGAAUGUGGUUCCAGTCAAGAGCTGGAACCACATTCAGTCCTTTTG-3’ |
|  | - | 5’-AATTCAAAAAGGACUGAAUGUGGUUCCAGCTCTTGACTGGAACCACATTCAGTCC-3’ |
| shVEGFA#1 | + | 5’-GATCCGTCACTAGCTTATCTTGAATCAAGAGTTCAAGATAAGCTAGTGACTTTTG-3’ |
|  | - | 5’-AATTCAAAAAGTCACTAGCTTATCTTGAACTCTTGATTCAAGATAAGCTAGTGACG-3’ |
| shVEGFA#2 | + | 5’-GATCCCTCTCTCCCTGATCGGTGATCAAGAGTCACCGATCAGGGAGAGAGTTTTTG-3’ |
|  | - | 5’-AATTCAAAAACTCTCTCCCTGATCGGTGACTCTTGATCACCGATCAGGGAGAGAGG-3’ |
| shcontrol | + | 5’-GATCCGAAGCCAGATCCAGCTTCCTCAAGAGGGAAGCTGGATCTGGCTTCTTTTTG-3’ |
|  | - | 5’-AATTCAAAAAGAAGCCAGATCCAGCTTCCCTCTTGAGGAAGCTGGATCTGGCTTCG-3’ |
