# Supplementary material for: Linc00511 acts as a competing endogenous RNA to regulate VEGFA expression through sponging hsa‐miR‐29b‐3p in pancreatic ductal adenocarcinoma
Source: J Cell Mol Med. 2017 Oct 5;22(1):655–67. doi: 10.1111/jcmm.13351 (PMC5742682; doi:10.1111/jcmm.13351)
Supplement: Supplementary file 2 — Data S1. Supplementary methods. [file JCMM-22-655-s002.docx]

**Supplementary Methods**

**Cell culture**

The human pancreatic cancer cell lines (PANC-1, MIA PaCa-2, Capan-2, SW1990, ASPC-1, BxPC-3) were purchased from the American Type Culture Collection (ATCC). An immortalized human pancreatic ductal epithelial cell line (HPDE6) was obtained from Dr. SN Zhang (Sun Yat-Sen University, Guangdong, China). All cells were cultivated in complete growth medium according to the manufacture, supplemented with 10% FBS and 1% penicillin/streptomycin. The cultures were maintained in a humidified 5% CO_2_ incubator at 37℃.

**RNA isolation and quantitative real-time PCR**

Total RNA was isolated from cells and tissues using TRIzol reagent (Invitrogen, San Diego, CA), and the purity of the total RNA was measured using the ratio of A260/A280 by NanoDrop. Then total RNA was converted to cDNA using M-MLV reverse transcriptase (Invitrogen). Oligo (dT18) RT primer was utilized for the reverse transcription of mRNA and lncRNA. Stem-loop RT primer was used for the reverse transcription of miR-497. For real-time quantitative PCR, three replicates per sample were amplified and analyzed with a Roche Light-Cycler (Roche, Basel, Switzerland). The qRT-PCR data were normalized using the endogenous GAPDH and U1 for linc00511. β-actin or U6 snRNA was used as internal controls for mRNA or miRNA, respectively. The relative gene expression in cells was determined using the comparative delta-delta CT method (2-∆∆Ct) and the fold change in gene expression of tissues was calculated using the standard ∆∆Ct method. All the primer sequences were provided in Table S1.

**Cell transfection and virus infection**

For transient knockdown experiments, SW1990 cells and ASPC-1 cells were seeded at a density of 1.5 ×10^5^ cells per well in 6-well plates. After 24 hours, cells were transfected with the following siRNAs: linc00511 siRNA#1, linc00511 siRNA#2 and scramble control siRNA, purchased from GenePharma Co (Shanghai, China), using Lipofectamine 3000 reagent (Invitrogen, San Diego, CA) in accordance with the manufacturer’s instructions. For lentivirus-mediated suppression of linc00511, the lentiviruses containing linc00511 were synthesized by GeneChem Co. Ltd (Shanghai, China). A non-targeting scrambled shRNA was also generated as a negative control. The shRNAs were inserted into the pMKO.1-puro vector. Lentiviral particles were obtained via transfecting 293T cells. Viral supernatants were collected 72 hours after transfection. For cell infection, SW1990 cells (5×10^5^ cells/well) were seeded in 6-well plates and infected with the lentiviral particles expressing linc00511 shRNA and scramble shRNA. All 72 hours later, puromycin (10ug/ml) was used to select puromycin-resistant clones. All oligonucleotide sequences are provided in Table S1.

**Western blotting**

Cells were washed in PBS and lysed with RIPA buffer (Invitrogen, Carlsbad, CA).For equal protein loading, a bicinchoninic acid protein assay kit (Pierce) was used to calculate protein concentration in each sample. Equivalent amounts of proteins were separated by SDS-PAGE and transferred to polyvinylidene fluoride (PVDF) membranes for immunoblotting. The membranes were blocked in 5% fat-free milk for 2 hours at room temperature, washed 3 times, and then the membranes were incubated with the following primary antibodies: mouse anti-human VEGFA polyclonal antibody (5ug/ml, #ab1316, Abcam, Cambridge, MA), mouse anti-human β-actin antibody (1:1000, #8226, Abcam, Cambridge, MA).β-actin was used as a loading control. They were then incubated with the following HRP-linked secondary antibody: goat anti-mouse IgG (1: 10000; Cell Signaling Technology, Boston, USA). An ECL chemiluminescence kit (Pierce) were used to detect bound antibody.

**Cell growth and cell apoptosis**

For cell growth assay, ASPC-1 or SW1990 cells with linc00511 knockdown were seeded in 96-well plates (1×103 cells per well) and pre-incubated at 37 ℃, 5% CO_2_ , in a humidified atmosphere for 0, 24, 48, 96 h. Subsequently, Counting Kit-8 kit (CCK-8) solution (10uL Dojindo Molecular Technologies, Kyushu, Japan) was added to each well and the plate was incubated for 4h at 37℃, 5% CO2, in a humidified atmosphere. The absorbance was measured at 450 nm using a microplate reader.

For cell death analysis, ASPC-1 or SW1990 cells (4×10^5^ cells) with linc00511 knockdown were trypsinized, washed twice with cold phosphate buffered saline (PBS), suspended in 400 ul of binding buffer, and divided into the control group and experiment group (200ul of each group). The experiment was treated with Annexin V-FITC reaction reagent containing 5ul of Annexin V-FITC and 10ul of PI. All the cells were placed in the dark for 10 min. Subsequently, the apoptosis was analyzed by flow cytometry.

**Wound-healing scrach assay and Boyden chamber cell invasion**

ASPC-1 and SW1990 cells were plated equally in 6-well plates and cultured in medium containing 10% FBS. After 24 hours, the cells were grew to reach almost total confluence. A wound scrach was created on the monolayer of cells with a 10ul pipette tip. Subsequently, phosphate-buffered saline was used to wash the cells. Images of the cells that had migrated into the wound were obtained at time point of 0h and 48h using a microscope (Nikon, Tokyo, Japan).

Invasion assays were performed using the BD Biocoat Matrigel Invasion Chamber (8um; BD Biosciences, San Jose, CA, USA) according to the manufacturer’ s protocol. After linc00511 silencing, 1×10^4^ cells were plated in the upper chamber. The bottom chamber contained medium with 10% FBS to stimulate invasion. After 48 hours, the bottom chamber insert was stained with 0.1% crystal violet, and cells were counted by photographing the memberane using a microscope and a × 20 objective lens. Each assay was conducted at least three times.

**Production of Conditioned Media (CM) and Tube Formation Assay**

Linc00511-downregulated ASPC-1 and SW1990 cells and their respective controls were cultured in DMEM containing 0.5% FBS. After 48 hours, conditioned media (CM) were collected, centrifuged at 1000rpm for ten minutes, filtered through a 0.22-um filter. ASPC-1 and SW1990 cells were also infected with shRNAs targeting VEGFA and control shRNA and their CM were harvested.

Matrigel (BD Bioscience) was thawed overnight at 4℃. A precooled 96-well plate was prepared with 75ul Matrigel in each well and then placed at 37 ℃ for 1 hour. HUVECs were incubated in CM for 24 hours and resuspended in respective CM. 100ul of the cell suspension (2×10^4^ cells) was loaded onto the surface of the polymerized Matrigel and incubated at 37 ℃ for 16 hours. Five randomly chosen fields were calculated and photographed (Olympus).

**RNA Binding Protein immunoprecipitation Assay**

RIP assay was conducted using the Magna RIP RNA-Binding Protein Immunoprecipitation Kit (Millipore, Bedford, MA, USA) according to the manufacturer’s protocol. Briefly, ASPC-1 cells and SW1990 cells were collected and lysed by RIP lysis buffer. Subsequently, 100ul cell extract was incubated with RIP buffer containing magnetic beads conjugated with human anti-Ago2 antibody or negative control normal mouse IgG. Proteinase K was used digest the protein and the immunoprecipitated RNA was purified. The isolated RNA was used for quantitative real-time PCR analysis of linc00511 and has-miR-29b-3p.

**Xenograft study**

All animal studies were approved by the institutional guidelines of Guangdong Province and by the Use Committee for Animal Care. All animal experiments were performed in accordance with both the institutional guidelines of Guangdong Province and United Kingdom Co-ordinating Committee on Cancer Research guidelines ([Workman et al, 2010](https://www.ncbi.nlm.nih.gov/pmc/articles/PMC4647875/#bib47))[20].We incubated subcutaneously in the flank area with SW1990 cells (4×10^6^ cells/mouse) stably infected with shRNA targeting linc00511 (six mice) or control shRNA (six mice). At day 5 after the injection of tumor cells, the tumors were evaluated every 3 days. The tumor volume was calculated (0.5×lenth×width^2^) and the mice were sacrificed at 20 days post incubation, and tumors were collected for further study (weight measurement, RNA extraction, and IHC). Briefly, tumor weights were measured in mice from the sh-linc00511(6 mice) or shcontrol (6 mice) groups. Linc00511 expression was evaluated by qRT-PCR, and tumor samples were excised and fixed in 4% paraformaldehyde solution for further IHC.

**Immunohistochemistry study**

Patient samples of primary carcinomas and the xenograft tumor specimens from nude mice were fixed in 4% paraformaldehyde and then embedded in paraffin. The 5μm sections were deparaffinized in xyleneand rehydrated in a graded series of ethanol, followed by heat-induced epitope retrieval in citrate buffer (pH6.0). Antigen retrieval was performed in 10 mmol/L citrate buffer (pH6.0) in a microwave oven for 15 min at 92~98℃. The activity of endogenous peroxidases was blocked by the addition of 3% hydrogen peroxide for 10 min at room temperature. Nonspecific staining was eliminated by 20min incubation with normal sheep serum (DAKO Corp. USA). The sections were incubated with rabbit anti-Ki67 antibody (1:125, #ab15580, Abcam) at 37℃for 2 h and then incubated with goat anti-rabbit secondary antibodies (#191866, Abcam, 0.2μg/ml) for 1h at 37ºC. The slides were incubated with a streptavidin-HRP conjugate complex for 45 min at 37°C. After rinsing three times in PBS, the sections were developed with 3,3-diaminobenzidine substrate. The nuclei were counterstained with hematoxylin. All images were captured via microscopy (Olympus). Ki67 staining was quantitated by determining the proportion of Ki67-positive cells. For VEGFA evaluation, we adopted a scoring criterion previously reported by Ohara et al. In brief, the staining intensity of VEGFA was graded on a scale of 0-3 (0, none; 1, weak; 2, intermediate; and 3, strong). HOXA13 expression was assessed according to the percentage of staining as follows: 0 points for none staining; 1 points for < 25% staining; 2 points for 26-50% staining; 3 points for 50-75% staining; 4 points for 50-75% staining. The total score was determined as the product of the scores for the intensity and positive rate of staining. Two pathologists assessed the staining following the scoring criteria. Cases with discrepancies were jointly reevaluated until a consensus was reached.
